# Impact of Oxalic Acid Consumption and pH on the In Vitro Biological Control of Oxalogenic Phytopathogen *Sclerotinia sclerotiorum*

**DOI:** 10.3390/jof11030191

**Published:** 2025-03-02

**Authors:** Aislinn Estoppey, Armelle Vallat-Michel, Patrick S. Chain, Saskia Bindschedler, Pilar Junier

**Affiliations:** 1 Laboratory of Microbiology, Institute of Biology, University of Neuchâtel, 2000 Neuchâtel, Switzerland; aislinn.estoppey@unine.ch (A.E.); saskia.bindschedler@unine.ch (S.B.); 2 Neuchâtel Platform of Analytical Chemistry, University of Neuchâtel, 2000 Neuchâtel, Switzerland; armelle.vallat@unine.ch; 3 Bioscience Division, Los Alamos National Laboratory, Los Alamos, NM 87545, USA; pchain@lanl.gov

**Keywords:** oxalotrophy, *Cupriavidus*, biocontrol, pH

## Abstract

The phytopathogenic fungus *Sclerotinia sclerotiorum* has a wide host range and causes significant economic losses in crops worldwide. This pathogen uses oxalic acid as a virulence factor; for this reason, the degradation of this organic acid by oxalotrophic bacteria has been proposed as a biological control approach. However, previous studies on the potential role of oxalotrophy in biocontrol did not investigate the differential effect of oxalic acid consumption and the subsequent pH alkalinisation on fungal growth. In this study, confrontation experiments on different media using a wild-type (WT) strain of *S. sclerotiorum* and an oxalate-deficient mutant (strain Δ*oah*) with the soil oxalotrophic bacteria *Cupriavidus necator* and *Cupriavidus oxalaticus* showed the combined effect of media composition on oxalic acid production, pH, and fungal growth control. Oxalotrophic bacteria were able to control *S. sclerotiorum* only in the medium in which oxalic acid was produced. However, the deficient Δ*oah* mutant was also controlled, indicating that the consumption of oxalic acid is not the sole mechanism of biocontrol. WT *S. sclerotiorum* acidified the medium when inoculated alone, while for both fungi, the pH of the medium changed from neutral to alkaline in the presence of bacteria. Therefore, medium alkalinisation independent of oxalotrophy contributes to fungal growth control.

## 1. Introduction

Humankind is dependent on crops and agricultural produce to feed an increasing global population. However, agriculture faces numerous challenges to achieve this, among which phytopathogenic fungi can be ranked as a major threat [1,2]. The intensive and systematic application of fungicides, which is the main control strategy currently employed, exerts a strong selection pressure on resistant pathogenic strains, making them even more difficult to control [2,3,4]. Therefore, it is necessary to develop and understand alternative pathogen control strategies to ensure future food security.

One of these strategies is biocontrol, which, in the case of the present study, will be defined as the deliberate use of a microorganism to control phytopathogenic fungi responsible for a crop disease [5,6]. The mechanisms underlying biocontrol are diverse and can range from competition to antibiosis. Moreover, the biocontrol agent can directly impair the growth of the pathogen, but it can also be beneficial by enhancing the host plant’s defences or even creating an environment that is not conducive to disease progression [6,7,8]. In the latter case, the biocontrol agent makes the environment suitable for itself at the expense of the pathogen. In 1998, Dickman and Chet [3] proposed the degradation of oxalic acid as a new approach to biological control. Oxalic acid (C_2_H_2_O_4_, pKa1 = 1.23, pKa2 = 4.19) is one of the many low-molecular-weight organic acids (LMWOA) produced by fungi and the strongest polyprotic acid after sulfuric acid. This organic acid is excreted by several pathogenic fungi as a pathogenicity factor [9,10,11,12]. The versatility of oxalic acid provides many advantages for fungal virulence: the acidification of the host tissues stimulates the production and activity of fungal enzymes. In addition, thanks to its chelating activity, oxalic acid can extract and sequester calcium ions from pectin, thus weakening the plant’s cell wall [9,13].

Oxalic acid is not only a factor in pathogenicity, but it is also one of the most commonly produced organic acids in the biogeosphere [14,15,16,17]. It plays a major role in the oxalate-carbonate pathway (OCP), a biogeochemical process acting as a potential sink for atmospheric CO_2_ in soils [18,19,20,21]. Oxalic acid enters the pathway mainly by the action of plants, which use it for the regulation and detoxification of calcium or for herbivory protection [22]. Organic matter decay by fungi releases oxalic acid in soils, provoking strong local acidification and precipitation of oxalate salts, mainly calcium oxalate [19,23]. Finally, oxalates are used as carbon and energy sources by oxalotrophic bacteria [14,16]. Oxalotrophic bacteria oxidise oxalic acid into CO_2_ and excrete it as bicarbonate ion HCO_3_^-^ [24,25,26,27]. As carbonic acid is a weak acid (pKa_1_ = 6.37 and pKa_2_ = 10.25), its secretion prompts local alkalinisation. When the pH reaches the equilibrium point for carbonic acid, calcium carbonate precipitation follows. This important local alkalinisation is the reason why calcium carbonate precipitation and accumulation occur. Thus, the OCP exerts a strong influence on soil pH and physicochemical properties [18,20,28].

**Figure 1:**
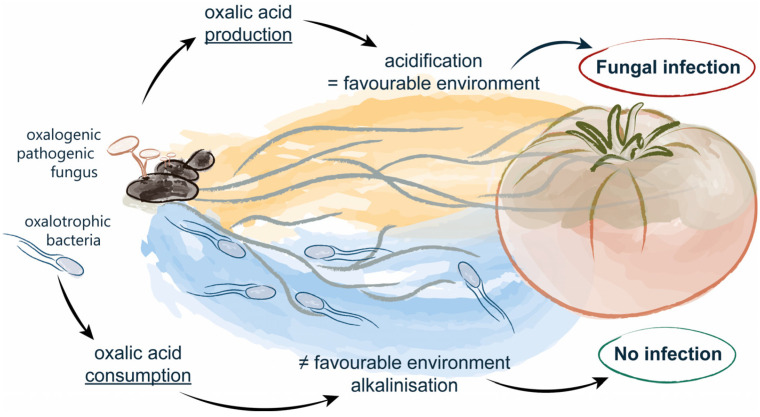
Schematic representation of the working hypothesis.

This propensity of the organisms involved in the OCP to modify the pH is the basis for the hypothesis of oxalotrophy as an effective biocontrol strategy that not only affects the pathogen, but also results in the modification of the local environment (Figure 1). Several studies have shown that oxalotrophic bacteria are indeed able to protect *Arabidopsis thaliana*, *Phaseolus vulgaris*, and other crops against *Sclerotinia sclerotiorum*, *Sclerotium rolfsii*, and *Botrytis cinerea*, three phytopathogenic fungi producing oxalic acid [3,29,30]. Both in vitro and field experiments have shown a significant reduction in disease incidence with the addition of oxalotrophic bacteria. However, none of these studies evaluated how the consumption of oxalic acid and the subsequent alkalinisation of the medium would interfere with fungal growth or how other nutritional factors could influence the interaction between oxalotrophic bacteria and the phytopathogenic fungus.

In order to disentangle the effect of oxalic acid consumption and alkalinisation on fungal growth, we investigated biocontrol of *S. sclerotiorum* and an oxalate-deficient mutant strain (Δ*oah S. sclerotiorum*) by oxalotrophic bacteria. This fungus is one of the most destructive necrotrophic pathogens, causing important losses in economically relevant crops [3,13,31,32]. Oxalic acid production has been extensively investigated for this pathogen, demonstrating that oxalic acid is essential for pathogenicity [31,33,34]. The oxalate-deficient mutant, Δ*oah S. sclerotiorum*, is unable to produce oxalic acid and is less virulent than the wild-type [35]. *Cupriavidus necator* and *Cupriavidus oxalaticus*, two facultative oxalotrophic bacteria from soil, were used as model bacteria for the interaction [36,37]. We monitored the biocontrol effect of *C. necator* and *C. oxalaticus* on two different media to investigate the influence of nutritional factors on the production of oxalic acid, changes in the pH, and the growth control of WT *S. sclerotiorum* and its mutant.

## 2. Materials and Methods

### 2.1. Fungi and Bacteria

The two strains of *S. sclerotiorum*, WT and Δ*oah* [35], were kindly provided by Jeffrey Rollins from the Department of Plant Pathology, University of Florida. The mutant was derived from a fully genome-sequenced wild-type *S. sclerotiorum* isolated from bean culls in western Nebraska. The bacteria *C. necator*-GFP [38] and *C. oxalaticus*-mCherry [37], bearing a fluorescent tag (GFP and mCherry, respectively), were obtained from the bacterial collection of the Laboratory of Microbiology, University of Neuchâtel, Neuchâtel, Switzerland. To improve the readability of the text, the GFP and mCherry suffixes were omitted from the text.

### 2.2. Fungal Sclerotia and Bacterial Suspensions

Sclerotia from WT *S. sclerotiorum* and Δ*oah* were harvested from 14-day-old fungal cultures grown on potato dextrose agar (PDA; 5 g/L potato infusion, 20 g/L dextrose, 17 g/L agar, pH 5.6 ± 0.2) and incubated at room temperature. Pure cultures of *C. necator* and *C. oxalaticus* were conserved in 30% glycerol at −80 °C, and suspensions were produced as described previously [39].

### 2.3. Soft Agar Confrontation Assays and Fungal Growth Area

Confrontation assays in soft agar were performed in triplicate in Reasoner’s 2A agar (R2A: 0.5 g/L yeast extract; 0.5 g/L peptone; 0.5 g/L casamino acids; 0.5 g/L glucose; 0.5 g/L soluble starch; 0.3 g/L Na-pyruvate; 0.3 g/L K_2_HPO_4_; 0.05 g/L MgSO_4_ · 7H_2_O; 15 g/L agar; in Milli-Q^®^ water [40]) and diluted malt agar medium (MA1/10: 1.2 g/L malt extract, 15 g/L agar) as described previously [39]. The fungal inoculum was a single sclerotium of WT or Δ*oah S. sclerotiorum* inoculated in the middle of the plate. The fungal growth area was calculated from the radius of the colony using pictures taken at 4 and 30 days post-inoculation [39].

### 2.4. Liquid Culture Confrontation, Oxalic Acid Concentration, and pH Measurements

Confrontation assays in liquid media were performed in triplicate in a 100 mm cell culture Petri dish [39]. The fungal inoculum was a single sclerotium of WT or Δ*oah S. sclerotiorum* placed in the middle of the plate. Oxalic acid concentration was determined by HPLC [39]. For pH measurements, readings were taken at the beginning and after 10 days of the experiment with a FiveEasy pH meter (Mettler Toledo, Greifensee, Switzerland). The initial pH of the media was 7.1 for R2B and 5.3 for MB1/10 (measured in triplicates). T-tests were used to compare the means of the different samples.

### 2.5. Images, Statistics, and Plotting of the Data

Macroscopic images were taken with a Nikon 1 V2 camera and processed (adjustment of white balance and cropping) with Adobe Photoshop CC 2018. Inverted microscopy images were taken with an EVOS M5000 microscope (Thermo Fisher Scientific, Waltham, MA, USA) and processed with ImageJ 2.0.0-rc65/1.51w to produce composite images of fluorescent and non-fluorescent samples. Figures were made using Adobe Illustrator CC 2018 and Adobe Photoshop CC 2018. Graphical figures and statistics were generated with GraphPad Prism 5.00.

## 3. Results

**Figure 2:**
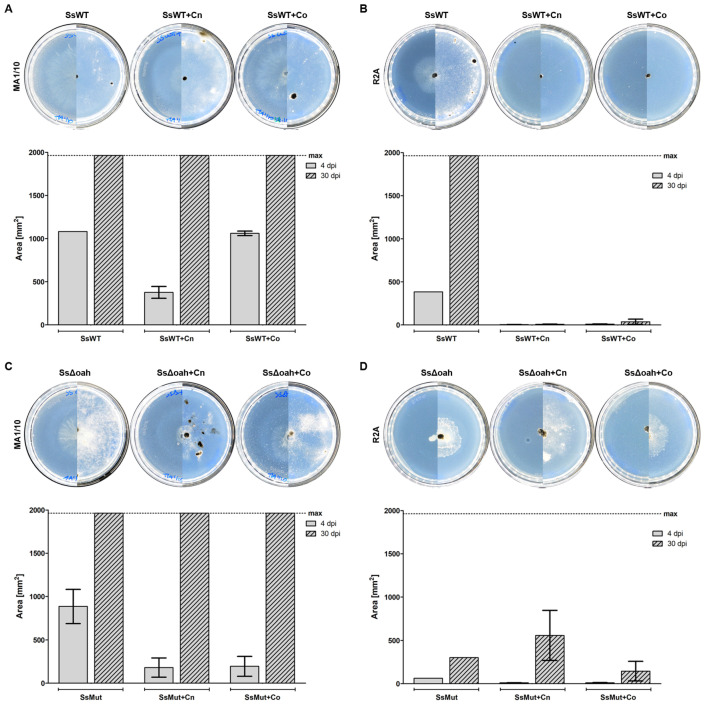
Soft agar confrontation assays between wild-type *Sclerotinia sclerotiorum* and the non-oxalogenic Δ*oah* mutant and oxalotrophic bacteria *Cupriavidus necator* and *Cupriavidus oxalaticus* on MA1/10 (**A**,**C**) and R2A (**B**,**D**). (**A**,**B**) From left to right: WT *S. sclerotiorum* (SsWT), WT *S. sclerotiorum* and *C. necator* (SsWT + Cn); WT *S. sclerotiorum* and *C. oxalaticus* (SsWT + Co). (**C**,**D**) From left to right: Δ*oah S. sclerotiorum* (SsΔoah); Δ*oah S. sclerotiorum* and *C. necator* (SsΔoah + Cn); Δ*oah S. sclerotiorum* and *C. oxalaticus* (SsΔoah + Co). The left half of the picture was taken after four days of incubation, and the right half—after 30 days of incubation. The area of mycelial growth was measured with ImageJ and plotted with the mean and the standard error of the mean in triplicates. After four days, the fungal growth area in the presence of *C. necator* or *C. oxalaticus* was significantly different from the control (*p*-values < 0.05) for both fungi on both media, while after 30 days, fungal growth was only significantly different on R2A (*p*-values < 0.05). The dotted line indicates the maximum area of the plate.

Confrontation assays were conducted with oxalogenic phytopathogenic fungus WT *S. sclerotiorum* and its mutant, Δ*oah S. sclerotiorum*, which is deficient in oxalic acid production. Both fungi were exposed to two oxalotrophic bacteria, *C. necator* and *C. oxalaticus*, on two media, MA1/10 and R2A. Sclerotia of the two fungal strains germinated and grew on the two media in the absence of the bacteria (Figure 2). However, the growth pattern changed in the presence of *C. necator* and *C. oxalaticus*. Mycelial growth was initially slowed down on MA1/10 in the presence of the oxalotrophic bacteria, but the presence of the bacteria did not control growth over time (Figure 2A,C). On the other hand, *C. necator* and *C. oxalaticus* exerted a strong growth control over WT and Δ*oah S. sclerotiorum* on R2A (Figure 2B,D). In the case of the Δ*oah* mutant, growth was hindered on R2A in the absence of bacteria, but the negative effect of *C. necator* and *C. oxalaticus* was still significant.

**Figure 3:**
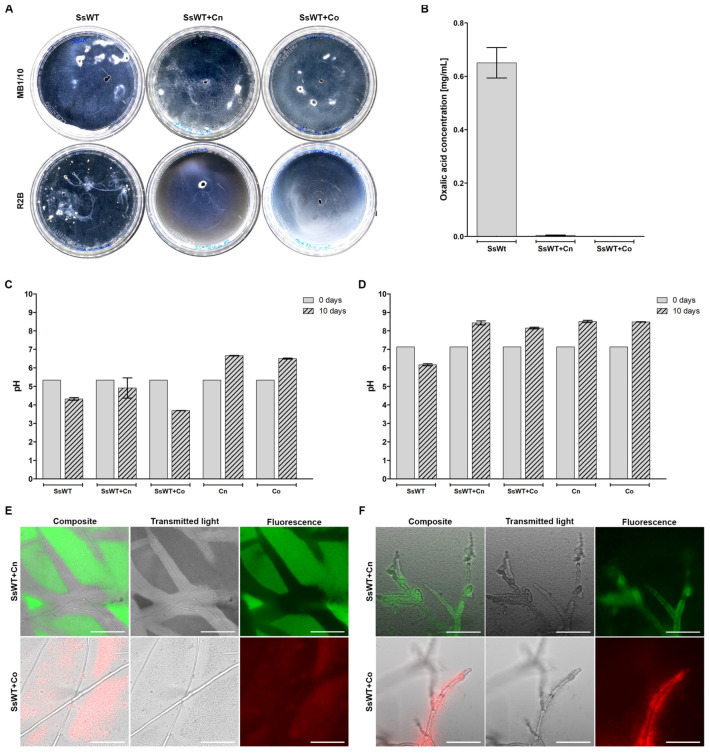
Confrontation assays in cell culture Petri dishes with WT *S. sclerotiorum*. (**A**) Images illustrating the growth on malt broth (MB1/10) on top and on Reasoner’s 2A broth (R2B) on the bottom; from left to right: WT *S. sclerotiorum* (SsWT); WT *S. sclerotiorum* and *C. necator* (SsWT + Cn); WT *S. sclerotiorum* and *C. oxalaticus* (SsWT + Co). (**B**) Corresponding oxalic acid detection in R2B. (**C**,**D**) Initial and final pH measurements in MB1/10 (**C**) and R2B (**D**). (**E**,**F**) Inverted microscopy images in MB1/10 (**E**) and R2B (**F**); scale bar = 100 µm. For oxalic acid concentrations (**B**), the mean of three independent measurements was plotted with the standard error of the mean. The oxalic acid concentration in the presence of *C. necator* and *C. oxalaticus* was significantly different from the control (*p*-values < 0.05). For pH measurements (**C**,**D**), the mean of three independent measurements taken at the beginning of the experiment and after 10 days was plotted with the mean and the standard error of the mean. After 10 days, the pH was significantly different from the initial pH in all cases except for WT *S. sclerotiorum* in the presence of *C. necator* in MB1/10 (**C**). The images acquired from the plates showed that *C. necator* and *C. oxalaticus* were alive after 10 days in both the MB1/10 and R2B media. The bacteria were found in large clumps away from the hyphae in MB1/10 (**E**), while they were closely associated with the WT *S. sclerotiorum* mycelium in R2B (**F**).

To quantify oxalic acid production and its impact on the pH of the medium, the same confrontation assays were conducted in cell culture Petri dishes with a thin layer of liquid versions of both media (MB1/10 and R2B). Sclerotia of WT *S. sclerotiorum* germinated and grew in both liquid media in the absence of the bacteria (Figure 3A). As in the confrontation assays on solid media, *C. necator* and *C. oxalaticus* were able to control the mycelial growth of WT *S. sclerotiorum*, but only in R2B (Figure 3A). Measurements of oxalic acid concentration showed that no oxalic acid was quantifiable in the MB1/10 medium, while its concentration reached 0.65 g/L in R2B after 10 days of incubation (Figure 3B). In R2B, oxalic acid production significantly decreased to 2 ± 3 mg/L in the presence of oxalotrophic bacteria (Figure 3B).

As expected, WT *S. sclerotiorum* significantly acidified the medium when inoculated alone. In MB1/10, the pH decreased from 5.3 to 4.3, while in R2B, it decreased from 7.1 to 6.2 (Figure 3C,D). However, this effect on the pH was drastically different in the presence of the oxalotrophic bacteria in both media. The final pH of the confrontation assays between WT *S. sclerotiorum* and the oxalotrophic bacteria in MB1/10 was significantly different from the initial pH, but not from the final pH of single cultures of WT *S. sclerotiorum* (Figure 3C). On the other hand, the final pH of the confrontation assays in R2B was significantly more alkaline than the initial pH or the pH of the control with fungi alone (Figure 3D). Interestingly, *C. necator* and *C. oxalaticus* significantly alkalinised both media in the absence of WT *S. sclerotiorum* (Figure 3C,D).

Bacterial viability was assessed by inverted microscopy on the plates based on their fluorescence tags (Figure 3E,F). *Cupriavidus necator* and *C. oxalaticus* were viable in all cases but showed a different behaviour depending on the medium used. In MB1/10, both bacteria were found in clumps organised away from the WT *S. sclerotiorum* mycelium (Figure 3E), while in R2B, the bacteria were closely associated with the hyphae (Figure 3F). The phenotype of the WT *Sclerotinia sclerotiorum* mycelium in R2B was also different from that in MB1/10 (Figure 3E,F). Fungal cells were enlarged and distorted in R2B, while in MB1/10, the hyphae were long and thin.

**Figure 4:**
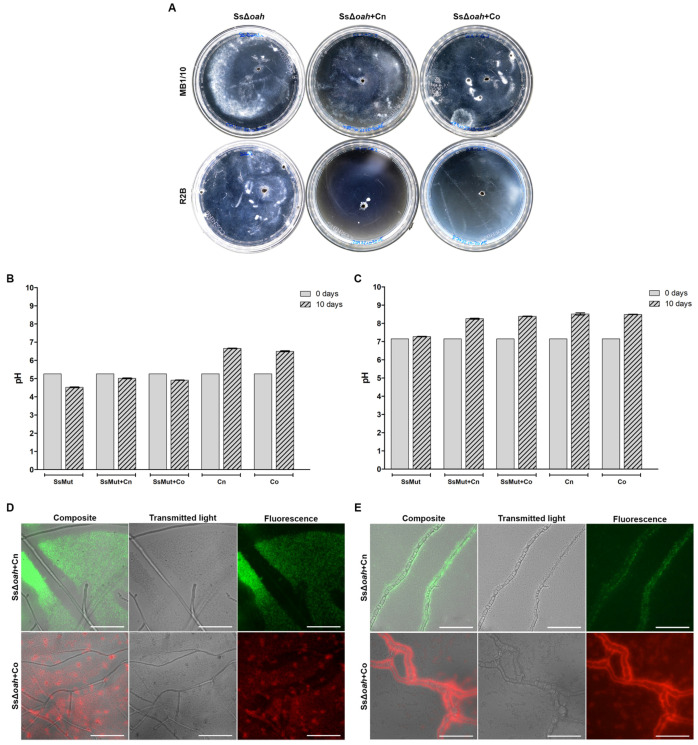
Confrontation assays in cell culture Petri dishes with Δ*oah S. sclerotiorum*. (**A**) Images illustrating the growth on malt broth (MB1/10) on top and on Reasoner’s 2A broth (R2B) on the bottom; from left to right: Δ*oah S. sclerotiorum* (SsΔoah); Δ*oah S. sclerotiorum* and *C. necator* (SsΔoah + Cn); Δ*oah S. sclerotiorum* and *C. oxalaticus* (SsΔoah + Co). (**B**,**C**) Initial and final pH measurements in MB1/10 (**C**) and R2B (**D**). (**D**,**E**) Inverted microscopy images in MB1/10 (**D**) and R2B (**E**); scale bar = 100 µm. For pH measurements (**B**,**C**), the mean of three independent measurements taken at the beginning of the experiment and after 10 days was plotted with the mean and the standard error of the mean. After 10 days, the pH was significantly different from the initial pH in all cases. The images acquired from the plates showed that *C. necator* and *C. oxalaticus* were alive after 10 days in both the MB1/10 and R2B media. The bacteria were found in large clumps away from the hyphae in MB1/10 (**D**), while they were closely associated with the Δ*oah S. sclerotiorum* Δ*oah* mycelium in R2B (**E**).

The same experiments were performed with the fungal mutant deficient in oxalic acid production. Sclerotia of Δ*oah S. sclerotiorum* germinated, and the fungus grew in both media in the absence of bacteria (Figure 4A). As expected, no oxalic acid was detected in the Δ*oah S. sclerotiorum*. However, Δ*oah S. sclerotiorum* significantly acidified the medium when cultured in MB1/10, both in the presence and absence of oxalotrophic bacteria (Figure 4B). On the other hand, Δ*oah S. sclerotiorum* did not acidify the R2B medium either alone or in the presence of *C. necator* and *C. oxalaticus* (Figure 4C). The pH of Δ*oah S. sclerotiorum* single cultures in R2B was slightly more alkaline than the initial pH, while it significantly changed from 7.1 to 8.4 in the presence of oxalotrophic bacteria. As with WT *S. sclerotiorum*, both bacteria were alive after 10 days and showed a similar behavioural pattern of interaction to the one observed with the wild-type fungus (Figure 4D,E).

## 4. Discussion

In order to assess the effect of oxalic acid consumption on fungal growth, the biocontrol effect of *C. necator* and *C. oxalaticus* on WT *S. sclerotiorum* and its oxalic acid-deficient mutant was monitored on two different media. In addition, as the production and consumption of oxalic acid are expected to have an impact on pH, this parameter was also monitored. Two different media were used to investigate the effect of nutritional factors on the interaction. The two media, MA1/10 and R2A, are commonly used for fungal or bacterial cultures, respectively. MA1/10 is a diluted version of the malt agar medium, and is mainly composed of oligosaccharides and amino acids. On the other hand, R2A contains various sources of carbon and amino acids, as well as potassium and magnesium sources [40]. As both media have a very different composition, oxalic acid production and growth varied. *Sclerotinia sclerotiorum* grew faster on MA1/10 than on R2A, while the oxalotrophic bacteria showed a clear growth preference for R2A compared to MA1/10. Moreover, *C. necator* and *C. oxalaticus* were able to control WT *S. sclerotiorum* only on R2A, which was the medium in which oxalic acid production was detected.

Even though oxalic acid was detected only in R2B, both the wild-type and mutant fungi were able to acidify MB1/10. Fungus *S. sclerotiorum* is known to produce not only oxalic acid, but also other LMWOAs, notably succinic, malic, and fumaric acids [41,42]. Most LMWOAs are produced through the same three pathways, namely the glyoxylate pathway, the reductive tricarboxylic acid cycle (TCA), and the oxidative TCA [42,43,44]. However, most studies appear to agree that these acids are produced mainly by a branch of the reductive TCA, the oxaloacetate pathway. After glycolysis, pyruvate or phosphoenolpyruvate is converted in oxaloacetate by the addition of CO_2_ and the use of ATP [44,45]. Oxaloacetate can then be converted into oxalate and acetate or into malate, and then further processed into fumarate and succinate or into citrate, to name a few [44,45,46]. Media composition plays a major role in the production of different LMWOAs as the medium has a direct impact on the efficiency of the enzymes involved in these pathways [42,47]. It has been shown, for example, that oxalic acid production is stimulated in media with a high nitrogen content, while fumaric acid production increases under nitrogen-limiting conditions [45,47]. This can explain the results obtained here, in which WT *S. sclerotiorum* produced oxalic acid only in the medium with a high nitrogen content (R2B). This appears to indicate that MB1/10 is not a suitable medium to study oxalic acid production/consumption as part of a biocontrol strategy. However, as no other LWMOA was detected by HPLC, the mechanism for the acidification in MB1/10 for both the wild-type and the mutant strains remains unclear. It is reported that Δ*oah Sclerotinia sclerotiorum* does not produce oxalic acid as it lacks one of the key genes required in the biosynthetic pathway [35]. Accordingly, this strain was not able to produce this or other LMWOAs on R2B and therefore failed to acidify this particular medium. This demonstrates the importance of the medium composition on the metabolism of the fungus, including LMWOA production.

As the biocontrol strategy evaluated here was hypothesized to be mediated through oxalic acid consumption, the lack of oxalic acid production in the MB1/10 medium is consistent with the lack of biocontrol in this particular medium. As bacterial consumption of oxalic acid and the subsequent carbonic acid production are the key factors promoting alkalinisation, the lack of oxalic acid production in MB1/10 would result in a failure of the biocontrol by oxalotrophic bacteria using this mechanism. However, the alkalinisation of both media when *C. necator* and *C. oxalaticus* were cultured alone indicates that oxalotrophy is not the only mechanism for alkalinisation. In fact, amino acid catabolism, peptone degradation, or different oxidative bacterial metabolisms can result in the generation of molecules that alkalinize the medium [36,48,49]. In particular, ammonia production can trigger a strong alkalinisation of the medium, potentially explaining changes in the pH in both media. However, as ammonia is a weak base, it is not able to counteract the constant acidification of the medium by *S. sclerotiorum* when in confrontation in MB1/10. On the other hand, the combined action of oxalic acid consumption and ammonia production on R2B would promote strong alkalinisation of this medium. Interestingly, Δ*oah S. sclerotiorum* was also controlled on R2B. As no oxalic acid was detected in the mutant’s spent medium, oxalic acid is clearly not the sole element involved in the biocontrol strategy investigated here.

The pH is in itself an important element to be considered. Previous studies in oxalotrophy as a biocontrol strategy did not investigate the effect of pH on the fitness of the fungus, but showed that the control occurred by a combination of mechanisms [3,29,30]. *Sclerotinia sclerotiorum* is known to acidify the environment to promote the production and activity of its enzymes and increase disease incidence [32,50,51]. Other oxalogenic fungi, such as *Aspergillus niger*, exhibit a wide array of mechanisms to maintain pH homeostasis in acidic environments. These fungal pathogens are adapted to thrive in acidic conditions, while alkaline pH appears to be highly stressful [52]. The effect of alkaline pH on *S. sclerotiorum* was dramatic, with reduced growth and modified hyphal morphology, possibly due to disrupted membrane integrity. Indeed, alkaline pH is known to trigger changes in membrane composition, in particular the production of glycosphingolipids, which ensure the formation of lipid rafts and the fluidity of the membrane. These changes are necessary to maintain an optimal cellular metabolism, and defects in sterol homeostasis result in the inability of the fungi to grow under alkaline conditions [53]. Here, the deformed hyphae of *S. sclerotiorum* observed in alkaline media could indicate defective signalling in membrane homeostasis, resulting in misshaped hyphae and reduced growth. In this case, the importance of the consumption of oxalic acid is lower, given the damage caused by the alkaline pH of the medium.

**Figure 5:**
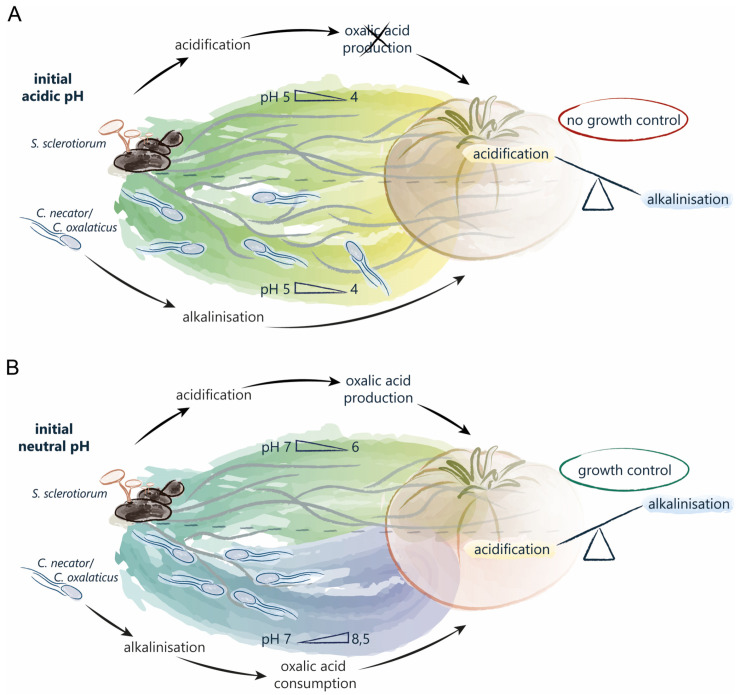
Schematic representation of the relationship between pH and fungal growth control by oxalotrophic bacteria when the initial pH is acidic (**A**) or neutral (**B**). With an acidic initial pH (**A**), *S. sclerotiorum* acidifies the medium but does not produce oxalic acid. Oxalotrophic bacterial metabolism is not sufficient to alkalinize the medium; therefore, fungal growth is not controlled. When the initial pH is neutral (**B**), *S. sclerotiorum* produces oxalic acid to acidify the medium. The combined action of oxalic acid consumption and bacterial metabolism promotes the alkalinisation of the medium and results in fungal growth control.

The initial pH of the medium also played a role in the growth rate of the pathogenic fungus. As an acidic pH is required for the production and activity of certain fungal enzymes [43,54], an initial acidic pH appears to be beneficial for *S. sclerotiorum*, while its growth is impaired at an alkaline pH. Both the mutant and the WT fungus grew faster on MA1/10 than on R2A. Since the initial pH of R2A is less favourable for *S. sclerotiorum*, its growth rate was reduced compared to MA1/10, where the initial pH is already in a range that is more favourable. On MA1/10 (pH 5.3), *S. sclerotiorum* does not need to produce oxalic acid but is able to simultaneously grow and acidify the medium, thus using nutritional resources in a more optimal way. *Cupriavidus necator* and *C. oxalaticus* metabolisms and the subsequent alkalinisation of the medium are not sufficient to tip over the pH from an acidic to an alkaline one (Figure 5A). In these conditions, *S. sclerotiorum* is more successful than the oxalotrophic bacteria, and was not controlled. On the other hand, on R2A (pH 7.1), *S. sclerotiorum* is already in a less favourable environment and first needs to acidify the medium. Thus, its growth is significantly slowed down and the oxalic acid produced is rapidly consumed by oxalotrophic bacteria. As *S. sclerotiorum* is unable to produce another LMWOA in this medium, the combination of oxalic acid consumption and oxidative bacterial metabolism induces a constant alkalinisation of the medium. In these conditions, *C. necator* and *C. oxalaticus* are more successful than *S. sclerotiorum* and can therefore control its growth (Figure 5B).

## 5. Conclusions

In conclusion, although oxalic acid consumption was initially proposed as the basis of the biocontrol strategy for oxalogenic fungi, we demonstrated here that the subsequent alkalinisation of the medium is as relevant for fungal growth control. The use of different media, as well as a fungus deficient in oxalic acid production, allowed us to disentangle the effect of the consumption of oxalic acid from that of alkalinization in the control of fungal growth. Regardless of the driver of alkalinisation (i.e., oxalic acid consumption or ammonia production), the pH change was ultimately responsible for the growth control of *S. sclerotiorum*. Future in planta studies evaluating, for instance, the role of ammonia or other mechanisms to control pH would be beneficial to translate this biocontrol strategy into praxis in agriculture.

## Data Availability

The original contributions presented in this study are included in the article. Further inquiries can be directed to the corresponding author.
